# Alterations of GPI transamidase subunits in head and neck squamous carcinoma

**DOI:** 10.1186/1476-4598-6-74

**Published:** 2007-11-21

**Authors:** Wei-Wen Jiang, Marianna Zahurak, Zeng-Tong Zhou, Hannah Lui Park, Zhong-Min Guo, Guo-Jun Wu, David Sidransky, Barry Trink, Joseph A Califano

**Affiliations:** 1 Department of Otolaryngology- Head and Neck Surgery, Head and Neck Cancer Research Division, Johns Hopkins Medical Institutions, 601 N. Caroline Street, 6th Floor, Baltimore, MD 21287-0910, USA; 2 Department of Oncology Biostatistics, Johns Hopkins Medical Institutions, Suite 1103, 550 North Broadway, Baltimore, 21205-2013, USA; 3 Department of Oral Medicine, The Ninth People's Hospital, School of Stomatology, Shanghai Jiao Tong University, 639 Zhizaoju Road, Huangpu District, Shanghai, Postcode 200011, China

## Abstract

**Background:**

GPI anchor attachment is catalyzed by the GPI transamidase (GPIT) complex. *GAA1, PIG-T *and *PIG-U *are the three of five GPIT subunits. Previous studies demonstrated amplification and overexpression of GPIT subunits in bladder and breast cancer with oncogenic function. We performed an analysis of these subunits in head and neck squamous cell carcinoma (HNSCC).

**Results:**

To evaluate *GAA1, PIG-T *and *PIG-U *in HNSCC, we used quantitative PCR (QPCR) and quantitative RT-PCR (QRT-PCR) to determine the copy number of those genes in primary tumors and the matching lymphocytes in 28 patients with HNSCC and quantified RNA expression of those genes in 16 primary HNSCC patients and 4 normal control tissue samples. *GAA1 *showed a significant increase in normalized mRNA expression, 2.11 (95% CI: 1.43, 2.79), in comparison to that of normal controls, 0.43 (95% CI: -0.76, 1.61), p = 0.014 (*Mann-Whitney test*). The mean genomic copy number of *GAA1 *was significantly increased in HNSCC, 0.59 (95% CI: 0.50, 0.79), in comparison to lymphocyte DNA, 0.35 (95% CI: 0.30, 0.50), p = 0.001 *(paired t-test)*.

**Conclusion:**

An increased expression level and elevated copy number for *GAA1 *suggest a role for this GPI anchor subunit in HNSCC.

## Background

HNSCC has an annual incidence of over than 40,000 cases per year in United States and is characterized by local tumor aggressiveness, a high rate of early recurrences, and development of second primary carcinomas [1]. Despite modern therapeutic strategies, overall 5-year survival rate does have only modestly improved.

Glycosylphosphatidylinositol (GPI) anchoring is a membrane attachment mechanism for cell surface proteins widely used in eukaryotes. GPI anchor attachment is catalyzed by GPI transamidase (GPIT) complex, which is composed of at least five subunits: *Phosphatidylinositol Glycan Class U (PIG-U), Glycosylphosphatidylinositol Anchor Attachment Protein 1 *(*GAA1*), *Phosphatidylinositol Glycan Class K (Gpi8), Phosphatidylinositol Glycan Class S (PIG-S), Phosphatidylinositol Glycan Class T (PIG-T) *[2-11]. All of the subunits are required for GPIT to function [3,6-8]. *Gpi8 *is the likely enzymatic component of the GPIT complex and can be cross-linked to proproteins [5,8,12-14]. *GAA1 *is able to assemble into *PIG-U*-containing GPIT complexes that are capable of interacting with a proprotein substrate, and this subunit also is critical in GPI recognition by GPIT [15]. *PIG-U *contains a sequence motif found in yeast and mammalian fatty acid elongases. This motif has been suggested to play a role in recognizing long chain fatty acids in GPI [3], and *PIG-T *contains endoplasmic reticulum (ER) localization information.

In a previous study amplification of *PIG-U *was noted in the bladder cancer, and was found to contribute to an oncogenic phenotype [16]. A recent study showed *PIG-T *and *GPAA1 *were overexpressed in breast cancer cell lines and primary tumors and caused malignant transformation *in vitro *[17]. Genomic amplification and/or DNA copy number gain are common genetic alterations in cancer that lead to the overexpression of oncogenes [18-20]. *PIG-U *and *PIG-T *are located at chromosome 20q and *GAA1 *is located at chromosome 8q, a chromosomal arm with increased copy number in HNSCC [21,22]. To evaluate *PIG-U, PIG-T and GAA1 *as possible oncogene candidates in HNSCC, we employed real-time PCR and real-time RT-PCR to determine copy number and RNA expression of those genes in primary HNSCCs.

## Results and discussion

### Analysis of mRNA expression of *GAA1*, *PIG-T *and *PIG-U *by QRT-PCR

**Figure 1:**
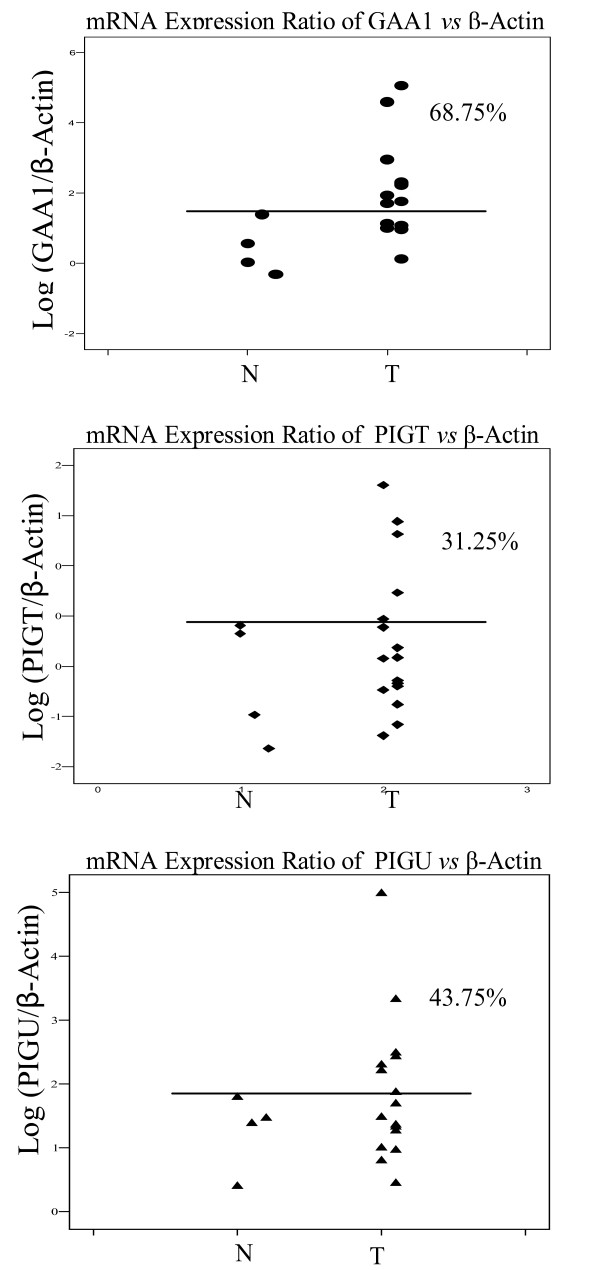
mRNA Expression Ratio of *GAA1*, *PIGT *and *PIGU vs β-Actin*. The expression differential of *GAA1*, *PIGT *and *PIGU *between normal (health subjects mucosa) and HNSCC was measured by calculating relative fluorescence amplification of transcripts of gene of interests normalized by the corresponding *β-Actin *signal. N, normal; T, HNSCC.

Overexpression of *PIG-U*, one of the GPIT subunits, was reported previously in primary bladder cancer and cell lines [16]. By comparing the mRNA expression ratios of the oral mucosa of normal subjects and tumor tissues of HNSCC patients, we were able to assess *GAA1, PIG-T *and *PIG-U *expression alteration in a quantitative manner. In this study, 68.75%, 31.25% and 43.75% of HNSCC exhibited higher mRNA expression in comparison to normal controls for *GAA1, PIG-T and PIG-U*, respectively (Fig 1). In particular, *GAA1 *showed a significant increase in mRNA expression in HNSCC, 2.11 (95% CI: 1.43, 2.79), in comparison to that in normal controls, 0.43 (95% CI: -0.76, 1.61), p = 0.014 (*Mann-Whitney test*).

### Analysis of copy number of *GAA1*, *PIG-T *and *PIG-U *by QPCR

**Figure 2:**
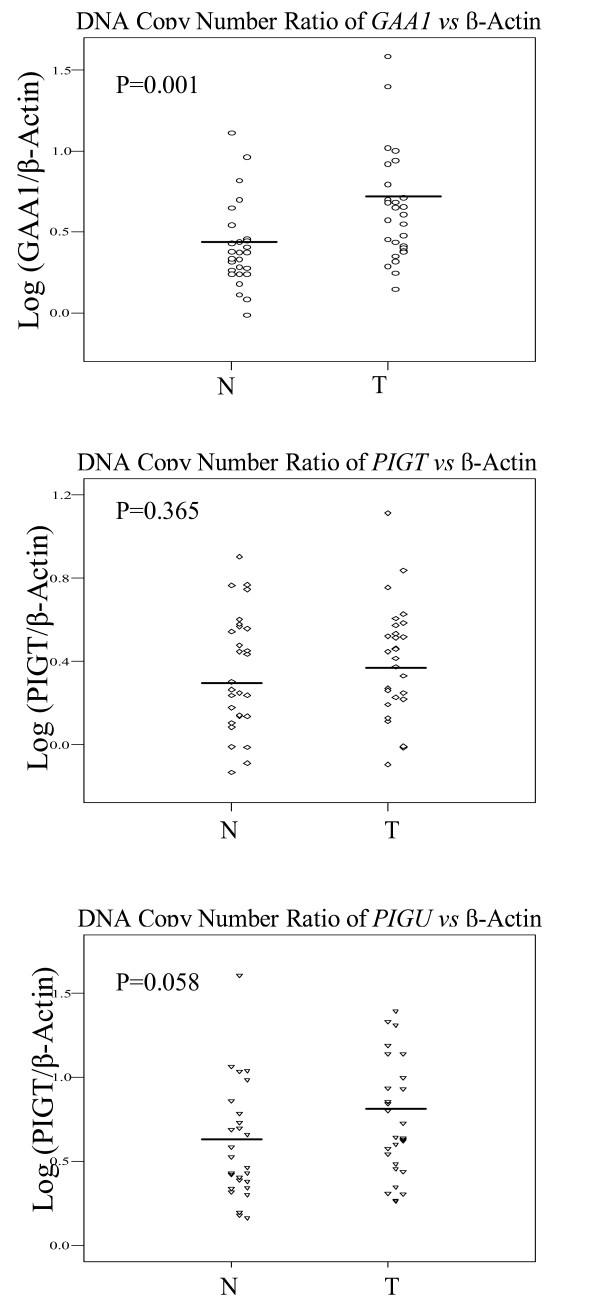
Relative DNA copy number of *GAA1*, *PIGT *and *PIGU *in HNSCC. The copy number differential of *GAA1, PIGT *and *PIGU *between paired lymphocytes and HNSCC was measured by calculating relative fluorescence amplification of gene of interest normalized by the corresponding *β-Actin *signal using standard curve method. N, normal; T, HNSCC.

The combination of QPCR and QRT-PCR is a facile approach to detecting gene amplification and over-expression in tumor specimens. To assess whether the copy number of *GAA1, PIG-T *and *PIG-U *was altered in HNSCC, we performed QPCR in 28 HNSCC and matched peripheral leukocyte DNA. The relative copy number of each gene *vs β-Actin *is shown in Table 1. Overall, a trend of increasing copy number was found in HNSCC. *GAA1 *copy number was significantly increased in primary HNSCCs, 0.59 (95% CI: 0.50, 0.79), in comparison to peripheral leukocyte DNA, 0.35 (95% CI: 0.30, 0.50), p = 0.001, while *PIG-U *showed a border line difference (*paired t-test*). (Fig 2) Taken together, the increased mRNA expression of *GAA1 *and low level copy number increase in HNSCC may be relevant as a possible contributor to oncogenic transformation in HNSCC.

**Table 1:** Copy number alteration of *GAA1, PIG-T *and *PIG-U *in HNSCC

DNA Copy Number Ratio		Number	Mean	Median	95%CI	*p (paired-t test)*
Log *(GAA1/β-Actin)*	Control	28	0.40	0.35	(0.30, 0.50)	0.001
	HNSCC	28	0.63	0.59	(0.50, 0.76)	
Log *(PIG-T/β-Actin)*	Control	28	0.34	0.28	(0.24, 0.45)	0.365
	HNSCC	28	0.40	0.43	(0.30, 0.50)	
Log *(PIG-U/β-Actin)*	Control	28	0.59	0.45	(0.46, 0.72)	0.058
	HNSCC	28	0.74	0.64	(0.61, 0.87)	

Many membranous enzymes, receptors, differentiation antigens and other biologically active proteins proved to be bound to the plasma membrane by GPI. Recent studies report that there are increased levels of the GPI-anchored adhesion molecules *CEACAM5*, *CEACAM6 *and mesothelin in response to forced expression of *ΔN-TCF-1B*, which could contribute to the induction of effecter molecules potentially relevant for tumor invasion in colorectal carcinomas patients [23]. Notably, our data showed that *GAA1 *copy numbers were much higher in HNSCC than in control white blood cells. This difference was highly significant, which was corresponded to the elevated RNA expression level in HNSCC patients.

### Clinical associations with copy number alterations in *GAA1*, *PIG-T *and *PIG-U*

To analyze whether clinical factors are associated with *GAA1, PIG-T *and *PIG-U *as potential markers, therefore, we further sub-grouped HNSCC patients by gender, race, stage and tumor site. We found that female HNSCC patients had a higher copy number of *GAA1*, 0.77 (95% CI: 0.63, 0.91), in comparison to male patients 0.58 (95% CI: 0.40, 0.75), p = 0.025 (*Mann-Whitney test*) (Table 2). In comparison to *GAA1*, *PIG-T *showed significant difference of copy number in paired lymphocytes DNA between male and female patients but not in tumor tissue DNA (Table 3b). Neither race nor tumor site was independently associated with *GAA1 *(Table 2), *PIG-T *(Table 3) and *PIG-U *(Table 4) copy number alterations. One might, therefore, speculate that gender might be a factor in the catalysis of GPI anchor attachment. Five out of 28 HNSCCs were located in larynx. We noticed that the *PIG-T *DNA copy number of laryngeal HNSCC, showed a relatively lower value in comparison to that in the other sites (Table 3), however, we were unable to draw definite conclusions due to limited case numbers. Additionally, no significances were shown among different stages for each gene.

**Table 2:** The effects of clinical parameters on copy number of *GAA1*

Log *(Gaa1/β-Actin)*		Number	Mean	Median	95%CI	*p (Mann-Whitney test)*
Control	Male	20	0.36	0.33	(0.25, 0.48)	0.242
	Female	8	0.49	0.39	(0.29, 0.70)	
HNSCC	Male	20	0.58	0.44	(0.40, 0.75)	0.025*
	Female	8	0.77	0.75	(0.63, 0.91)	
Control	White	17	0.35	0.32	(0.23, 0.46)	0.165
	Other	11	0.48	0.43	(0.29, 0.68)	
HNSCC	White	17	0.52	0.48	(0.42, 0.63)	0.095
	Other	11	0.81	0.68	(0.52, 1.09)	
Control	OC	11	0.35	0.33	(0.18, 0.53)	
	HP	3	0.46	0.43	(0.05, 0.88)	0.392
	L	5	0.42	0.28	(0.04, 0.80)	0.865
	OP	9	0.43	0.38	(0.22, 0.64)	0.518
HNSCC	OC	11	0.67	0.68	(0.39, 0.94)	
	HP	3	0.55	0.11	(-0.26, 1.37)	0.697
	L	5	0.65	0.61	(0.37, 0.92)	0.955
	OP	9	0.61	0.57	(0.36, 0.86)	0.676

**Table 3:** The effects of clinical parameters on copy number of *PIG-T*

Log *(PIG-T/β-Actin)*		Number	Mean	Median	95%CI	*p (Mann-Whitney test)*
Control	Male	20	0.28	0.25	(0.15, 0.41)	0.042*
	Female	8	0.51	0.56	(0.30, 0.72)	
HNSCC	Male	20	0.37	0.39	(0.24, 0.49)	0.222
	Female	8	0.48	0.54	(0.25, 0.71)	
Control	White	17	0.33	0.44	(0.18, 0.47)	0.621
	Other	11	0.37	0.26	(0.18, 0.57)	
HNSCC	White	17	0.40	0.45	(0.29, 0.51)	0.621
	Other	11	0.40	0.41	(0.16, 0.63)	
Control	OC	11	0.39	0.43	(0.18, 0.61)	
	HP	3	0.46	0.54	(-0.40, 1.33)	0.938
	L	5	0.35	0.25	(-0.01, 0.72)	0.692
	OP	9	0.24	0.24	(0.08, 0.40)	0.239
HNSCC	OC	11	0.52	0.52	(0.34, 0.70)	
	HP	3	0.44	0.10	(-0.34, 1.22)	0.586
	L	5	0.20	0.19	(-0.08, 0.48)	0.027*
	OP	9	0.35	0.41	(0.17, 0.53)	0.271

**Table 4:** The effects of clinical parameters on copy number of *PIG-U*

Log *(PIG-U/β-Actin)*		Number	Mean	Median	95%CI	*p (Mann-Whitney test)*
Control	Male	20	0.56	0.43	(0.39, 0.72)	0.476
	Female	8	0.66	0.68	(0.41, 0.92)	
HNSCC	Male	20	0.65	0.62	(0.51, 0.80)	0.053
	Female	8	0.95	0.97	(0.66, 1.24)	
Control	White	17	0.56	0.43	(0.40, 0.71)	0.655
	Other	11	0.64	0.59	(0.38, 0.89)	
HNSCC	White	17	0.69	0.68	(0.53, 0.86)	0.466
	Other	11	0.81	0.64	(0.56, 1.06)	
Control	OC	11	0.57	0.41	(0.29, 0.86)	
	HP	3	0.57	0.43	(-0.05, 1.19)	0.484
	L	5	0.62	0.59	(0.27, 0.96)	0.533
	OP	9	0.59	0.43	(0.36, 0.82)	0.470
HNSCC	OC	11	0.83	0.80	(0.57, 0.10)	
	HP	3	0.68	0.54	(-0.45, 1.81)	0.586
	L	5	0.67	0.57	(0.19, 1.16)	0.396
	OP	9	0.69	0.64	(0.51, 0.86)	0.425

## Conclusion

Our data indicate that increasing of copy number and mRNA expression of *GAA1 *is characteristic for HNSCC and *GAA1 *play a role for this GPI anchor subunit in HNSCC.

## Methods

### Subjects

Genomic DNA samples from 28 HNSCC tissues and matching lymphocytes were subjected to QPCR analysis. RNA of tissue samples from 16 microdissected HNSCCs and 4 oral mucosal biopsies of healthy people were subjected to QRT-PCR. All samples were collected at the Department of Otolaryngology-Head and Neck Surgery, School of Medicine, The Johns Hopkins University, after appropriate approval was obtained from the Johns Hopkins institutional review board (IRB approval # **03-04-11-131d**).

### Cell lines and culture conditions

DNA and RNA from a virally transformed human cutaneous keratinocyte cell line (HaCaT) were used as standards for QPCR and QRT-PCR. The HaCaT cell line was grown in DMEM media supplemented with 10% FBS and 1% Penicillin-Streptomycin. Media components were obtained from Gibco Invitrogen Corporation (Carlsbad, CA) and cells were incubated at 37°C in an atmosphere of 5% CO_2_/95% relative humidity.

### Quantitative PCR

A Perkin-Elmer/ABI 7900 thermocycler (Applied Biosystems, CA) was used to perform QPCR amplification for *β-actin*, *PIG-U, PIG-T *and *Gaa1*. *β-actin *primer and probe sequences used previously were employed in this study [24]. *PIG-U*[16], *PIG-T *and *GAA1 *primer and probe sequences were listed in Table 5. Primers were custom made and obtained from Invitrogen (Carlsbad, CA). All TaqMan probes (Applied Biosystems, Foster City, CA) were 5'-FAM and 3'-TAMRA labeled. PCR amplifications were carried out in buffer containing: 16.6 mM ammonium sulfate, 67 mM Trizma (pH 8.8), 2.5 mM MgCl_2_, 10 mM β-mercaptoethanol, 0.1% DMSO, 600 nM each of forward and reverse primers, 200 nM TaqMan probe, 0.6 units Platinum Taq polymerase, and 2% ROX reference dye. 500 picograms of DNA were used to amplify the mitochondrial regions whereas 10 ng were used to amplify *β-actin*. The real-time PCR reactions were performed in triplicate for each gene. Data analysis was performed using Microsoft Excel software.*PIG-U, PIG-T *and *GAA1 *were normalized using the corresponding *β-actin *signal.

**Table 5:** *PIG-U, PIG-T *and *GAA1 *primer and probe sequences

Gene	Forward Primer	Probe	Reverse Primer
*PIG-U*	AGCCCTCCAGCCAGAGTTA	CAGGCGAGTGCTTGGGCAGAAGA	ACTTGTGACCCTGGACTCGAA
*PIG-T*	GATCTGCCTCACGTGCACTGT	TGGCCGTGTGCTATGGCTCCTTC	AGGTTCGGGTGAGGAGATTGT
*Gaa1*	CCGGGCTGGGACAGAGA	TCCCCAAGGACCCCATTCTGCC	CAGACACTCATTTATTTCCCCA

### Statistical analysis

The major statistical first endpoint in this study was the comparison of the difference in *PIG-U, PIG-T *and *GAA1 *quantitative DNA ratio between lymphocytes DNA and tumor tissue DNA in the HNSCC patients. The second endpoint in this study was *PIG-U, PIG-T *and *GAA1 *RNA expression differences between HNSCC patients and normal control subjects. Distributions of *PIG-U, PIG-T *and *GAA1 *DNA and RNA quantitative ratios were examined graphically using scatter plots and bar plots with logarithmic scales. Log transformation was chosen for these values and the difference taken for statistical analyses. Paired *t-tests *were used to determine if these changes were significantly different from each other. All statistical computations were performed using the SPSS system (SPSS, Chicago) and all p values reported are two-sided.

## Authors' contributions

WWJ carried out cell cultures, QRT-PCR, QPCR, conceived the study, participated in its design, and drafted the manuscript. MZ analyzed the data and performed the statistical analysis. ZTZ and HLP critically revised the manuscript. WGJ and ZMG participated in the design of the study. DS conceived the study. BT conceived the study, participated in its design. JAC conceived the study, participated in its design and coordination, and contributed to the final drafting and critical revision of the manuscript. All authors read and approved the final manuscript.
